# An Active Inference Model of the Optimism Bias

**DOI:** 10.5334/cpsy.125

**Published:** 2025-01-31

**Authors:** Elizabeth L. Fisher, Christopher J. Whyte, Jakob Hohwy

**Affiliations:** 1 Monash Centre for Consciousness and Contemplative Studies, Monash University, Melbourne, Australia; 2 Brain and Mind Centre, The University of Sydney, Sydney, Australia; 3 Centre for Complex Systems, The University of Sydney, Sydney, Australia

**Keywords:** optimism bias, active inference, belief updating, depression

## Abstract

The optimism bias is a cognitive bias where individuals overestimate the likelihood of good outcomes and underestimate the likelihood of bad outcomes. Associated with improved quality of life, optimism bias is considered to be adaptive and is a promising avenue of research for mental health interventions in conditions where individuals lack optimism such as major depressive disorder. Here we lay the groundwork for future research on optimism as an intervention by introducing a domain general formal model of optimism bias, which can be applied in different task settings. Employing the active inference framework, we propose a model of the optimism bias as high precision likelihood biased towards positive outcomes. First, we simulate how optimism may be lost during development by exposure to negative events. We then ground our model in the empirical literature by showing how the developmentally acquired differences in optimism are expressed in a belief updating task typically used to assess optimism bias. Finally, we show how optimism affects action in a modified two-armed bandit task. Our model and the simulations it affords provide a computational basis for understanding how optimism bias may emerge, how it may be expressed in standard tasks used to assess optimism, and how it affects agents’ decision-making and actions; in combination, this provides a basis for future research on optimism as a mental health intervention.

## Introduction

The optimism bias is cognitive bias where individuals overestimate the likelihood of good outcomes and underestimate the likelihood of bad outcomes (Sharot, 2011; Sharot et al., 2007). Studies of the optimism bias suggest optimism is adaptive, as it is associated with improved quality of life (Sharot, 2011). Benefits related to optimism include lower risk of all-cause mortality and cardiovascular disease (Krittanawong et al., 2022), engagement in more self-care behaviours and lower fatigue symptoms reported in chronic illness (de Ridder et al., 2004), improved cognitive functioning following traumatic brain injury (Lee et al., 2019), improved cardiovascular response to stress (Terrill et al., 2010), and immune function, where optimists have improved immune response following a stressor (Curzytek et al., 2018). Social factors that are improved by optimism include higher salaries at work, greater achievement in education, and better performance in sport (Sharot, 2011). Taking these findings together, it is unsurprising that optimistic individuals have less overall stress and worry (Conversano et al., 2010). Interestingly, optimism is a robust bias and is seen across species (Bateson et al., 2011; Iki & Adachi, 2023; Lagisz et al., 2020). However, mental health conditions, such as major depression, are linked to diminished optimism or a negativity bias suggesting optimism may be lost throughout life (Garrett et al., 2014; Korn et al., 2014). Specifically, individuals suffering from depression have been shown to either have no optimism bias, or a pessimism bias where individuals overestimate the likelihood of negative outcomes (Garrett et al., 2014; Hobbs et al., 2022; Korn et al., 2014; Kube & Rozenkrantz, 2021). Core symptoms of depression, such as anhedonia and apathy result in withdrawing from the world, the opposite behaviour that optimism promotes (Cooper et al., 2018; Fisher et al., 2024; Rizvi et al., 2016), and treatment of depression includes behavioural activation that reverses this behaviour by encouraging engagement with the world (Soucy Chartier & Provencher, 2013; Tindall et al., 2017).

Given the multitude of beneficial outcomes related to the optimism bias, and its propensity to increase one’s engagement with the world in line with behavioural activation treatments, this bias may offer a promising avenue of research into interventions for mental health conditions in cases where individuals appear to lack optimism or have a negativity bias. To consider the optimism bias as a potential target for mental health interventions, it is important to have a grounding quantitative understanding of how it can be acquired over the course of development, how it is expressed in common tasks designed to measure it, and how it affects actions. Here, we propose a computational model of optimism bias and use a series of simulation experiments to deepen our understanding of optimism bias across these domains. This quantitative understanding of optimism allows us to approach a number of questions relevant to treatment such as the level of optimism most conducive for positive outcomes, who may benefit from an optimism bias intervention, and potential unwanted side effects of increasing optimism. The aspirational end goal in developing this computational model is to explore treatments tailored to individual patients, taking into account their particular symptoms and environment (Adams et al., 2015; Friston, 2022; Goldway et al., 2023; Huys et al., 2016; Montague et al., 2012).

To provide a formal conceptualisation of the optimism bias, we employ the active inference framework (Friston & Stephan, 2007; Parr et al., 2022; Smith et al., 2022) to propose a computational model of optimism bias. We model optimism bias as a domain general prior belief of positive outcomes being more probable than negative outcomes; this is the belief ‘I believe I will observe a good outcome’. A high precision likelihood biased towards positive outcomes is implemented by the likelihood matrix, where the likelihood matrix is conditioned on the optimism bias state factor; this means that under the optimistic hidden state the likelihood mapping is more precise for good outcomes. A high precision likelihood biased towards positive outcomes results in agents updating their beliefs more to positive observations than negative observations and thereby developing the asymmetry in belief updating characteristic of the optimism bias (Bottemanne et al., 2022; Garrett et al., 2014; Sharot et al., 2007). A domain general model of the optimism bias allows us to apply the model to a range of different task settings through simulation, speaking to the rich literature on the bias. We begin by showing how optimism bias may be lost and acquired in development, taking arousal into account. We then leverage a belief updating task to show how our model corresponds to studies of levels of optimism, as found in the literature. Finally, we use a modified two-armed bandit task to show how optimism affects action in a task setting. By developing an optimism bias model that demonstrates how optimism is related to development, belief updating, and action, we provide a foundation for further research into optimism as a potential intervention.

## Methods

### The active inference framework

The active inference framework models perception, planning and decision making as hidden state inference, and learning as parameter estimation, under a generative model of the environment (Friston & Stephan, 2007; Parr et al., 2022; Sajid et al., 2021; Smith et al., 2022). The generative model takes the form of a partially observable Markov decision process (Littman, 2009), which models perception and decision making in environments where there is an uncertain mapping between the hidden states (*s*) generating sensory observations and the sensory observations themselves (*o*). The full joint probability for POMDP under a specific policy is shown below where *p*(*s*_1_) is the prior over the initial state of the agent, *π* is the policy of the agent, *p*(*o_τ_*|*s_τ_*) the likelihood, and *p*(s*_τ_*│(s*_τ_*_–1_, *π*) the transition probability.

\[p\left({{o_{1:{\mathrm{T}}}}{\mathrm{}},\,\,{s_{1:{\mathrm{T}}}}{\mathrm{|\pi }}} \right) = {\mathrm{}}p({s_1}){\mathrm{}}\mathop \prod \limits_{\tau = 1}^{\mathrm{T}} p({o_\tau}|{s_\tau})\mathop \prod \limits_{\tau = 2}^{\mathrm{T}} p({s_\tau }|{s_{\tau - 1}},\,\,\pi)\]

Perception and decision making under this model proceed in a two-step fashion. For the first, perceptual step, the agent must invert the generative model to infer the posterior probability of hidden states given a set of observations. Under active inference this is formulated as variational Bayesian inference, which infers an approximate posterior distribution *q*(*s_τ_*|*π*) = ***s***_*π,*_*_τ_* (where bolded vectors denote a posterior distribution) through the minimisation of marginal free energy (*F*), an objective function encoding the expected difference between the approximate posterior and generative model (the POMDP). The equation for marginal free energy is shown below in matrix form where (**A** = *p*(*o_τ_*│*s_τ_*)) is a likelihood matrix encoding how likely an observation is given the state an agent is in. The transition matrix, (**B** = *p*(*s_τ_*│*s_τ_*_–1_, *π*)) encodes how the hidden states change over time (that is, given the agent was in one state at time 1, what is the probability they are in that state at time 2). **B**^†^ denotes the transpose of **B** with normalised columns (i.e., columns that sum to 1) and *o_τ_* is a one hot vector encoding the observations generated by the generative process at each time step.

\[{F_{\pi,\tau}}\, = {s_{\pi,\tau}} \cdot ({\mathrm{ln}}{s_{\pi,\tau}} - \frac{{1}}{2}({\mathrm{ln}}\,\,{{\bf{B}}_{\pi,\tau - 1}}{s_{\pi,\tau - 1}} + {\mathrm{ln}}\,\,{{\bf{B}}^\dagger}_{\pi,\tau }{s_{\pi,\tau + 1}}) - \ln \,\,{{\bf{A}}^{\mathrm{T}}}\,\,{o_\tau})\]

The approximate posterior is iteratively updated through gradient descent where \[ - {\nabla _{{s_{\pi,\tau }}}}{F_\pi } = {\varepsilon _{\pi,\tau}}\] until the model converges (in practice for relatively simple models, such as those used in this paper, the posterior converges rapidly so we only consider a maximum of 16 steps). The posterior distribution over states (***s***_*π,*_*_τ_*) updates with each iteration. The update equations describing this process are shown below where *σ*(∙) denotes the softmax function:

\[{\varepsilon _{\pi,\tau }} \leftarrow \ln \,\,{{\bf{A}}^{\mathrm{T}}}\,\,\,{o_\tau } + \frac{1}{2}\left({\ln \left({{{\bf{B}}_{\pi \tau }}{s_{\pi \tau - 1}}} \right)\,\, + \,\,\ln \left({{{\bf{B}}_{\pi \tau }}{s_{\pi \tau - 1}}} \right)} \right) - \ln\,{s_{\pi,\tau }}\]

\[{v_{\pi,\tau }} \leftarrow {v_{\pi,\tau }} + {\varepsilon _{\pi,\tau }}\]

\[{s_{\pi,\tau }} \leftarrow \sigma \left({{v_{\pi,\tau }}} \right)\]

As the state of the environment depends on the actions of the agent (e.g. the location of the eyes in a visual field), the inference procedure for hidden states is conditioned on each policy (*q*(*s_τ_*|*π*) = ***s***_*π,*_*_τ_*). This inference procedure equips the agent with a posterior distribution ***s***_*π,*_*_τ_* approximating the true state of the environment under each action.

Next the agent must infer the posterior over policies (*q*(*π*) = ***π*** = *σ*(*G*_*π*_)) (action sequences) that will minimise expected free energy (*G*), which encodes the expected difference between the approximate posterior distribution and the generative model. Note that for the decision-making step, observations must be treated as random variables as future observations by definition are not yet known by the agent (where ***As***_*π,*_*_τ_* = ***o***_*π,*_*_τ_*) denote the observations expected under each policy).

\[{G_\pi } = \mathop \sum \limits_\tau \left({\underbrace {{\bf{As}}_{\pi,\tau} \cdot \left({\ln {\bf{As}}_{\pi,\tau} - \ln {{\bf{C}}_\tau}} \right)}_{risk} - \underbrace {diag\left({{{\bf{A}}^{\mathrm{T}}}\ln {\bf{A}}} \right) \cdot {{\bf s}_{\pi,\tau}}}_{ambiguity} - \underbrace {{\bf{As}}_{\pi,\tau} \cdot {\bf{Ws}}_{\pi,\tau}}_{novelty}} \right)\]

\[{\bf{W}}:\, = \frac{{1}}{2}(a{^{{\odot}(-1)}} - a_{sums}^{{\odot}(-1)})\]

To minimise expected free energy (*G*_*π*_) the agent must select policies which maximise the probability of receiving preferred observations (i.e., minimise risk), reduce uncertainty between hidden states and observations (i.e., minimise ambiguity), and minimise uncertainty in the parameters of the generative model (i.e., maximise novelty). The first term in *G*_*π*_ risk, scores how well the observations expected under each policy align with the agent’s prior preferences encoded in the matrix **C**. The second term, ambiguity, scores the precision of the mapping between the hidden states and observations expected under each policy. The third term (**A*s***_*π,*_*_τ_*⋅**W*s***_*π,*_*_τ_*) novelty quantifies the extent to which the observations expected under each policy increase the agent’s certainty about the parameters of the likelihood matrix, which drives the agent to seek novel information. Here ⨀ denotes the element-wise power, ***a*** is a matrix containing the concentration parameters of **A**, and ***a***_*sums*_ is a matrix the same size as ***a*** whose entries encode the sum of the columns in ***a***. The entries of ***a*** are updated on each trial by the learning rule described below. The function of this term can be understood heuristically by noticing that when agents have extensive exposure to a set of observations ***a*** and ***a***_*sums*_ have large values so the **W** term, which holds the inverse values of ***a*** and ***a***_*sums*_, is small, generating a small novelty value. In contrast, when agents have no experience with a set of observations the concentration parameters are small so the **W** term is large.

Under active inference, learning involves updating the concentration parameters of the (prior) Dirichlet distributions over the categorical distributions encoded in the **D** vector and **A** and **B** matrices. For **A** matrix learning, a count representing the co-occurrence of a state-observation pair is added after every observation, and for **D** vector learning a count representing the state the agent found itself in is added to the parameters of the **D** vector. The relevant equations are shown below where ***s****_τ_* = ∑***πs***_*π,*_*_τ_* denotes the Bayesian model average over policies.

\[p({\bf{A}})\,\, = \,\,Dirichlet(a)\]

\[a\,\, = \,\,\left[ {\begin{array}{*{20}{c}}
{{a_1}}&{{a_2}}&{{a_3}}\\
{{a_4}}&{{a_5}}&{{a_6}}\\
{{a_7}}&{{a_8}}&{{a_9}}
\end{array}} \right]\]

\[{a_{trial + 1}} = {a_{trial}} + \mathop \sum \limits_t {o_{t}} \otimes {s_t}\]

\[p({\bf{D}})\,\, = \,\,Dirichlet(d)\]

\[d = {[{d_1}\,{d_2}]^{\mathrm{T}}}\]

\[{d_{trial + 1}} = {d_{trial}} + \,{s_{\tau = 1}}\]

We have so far described the generative model capturing the agents’ beliefs about the environment. The environment itself is captured by the generative process, which in this case is identical in structure to the generative model (i.e., shares the same **A, B, D** matrices) but can differ in terms of the parameters of the probability distributions, and does not include parameter learning or policy selection. For example, the generative process of a gambling task might have the objective probability of a win in the left arm at 70%, but the agent may believe that it is more or less than this value. This aspect of the active inference framework is crucial to our model of optimism bias which, by definition, involves agents overestimating the likelihood of good events. We denote vectors and matrices encoding the generative process when it is distinct from the generative model with an accent (e.g., **Â** denotes the likelihood of the generative process and **a** the generative model).

Finally, we note that in principle, when formulated as a POMDP, the active inference framework is equivalent to variants of model-based reinforcement learning that include information seeking terms that maximise the information gain about the mapping between hidden states and observations (e.g., Houthooft et al., 2017) and maximise parameter novelty (e.g. UCB (Sutton & Barto, 2018)). We opted to use active inference for reasons of convenience as it is not necessary to include ad hoc terms in the objective function, and for congruence with a previous study, which used active inference models to fit to behavioural data on optimism bias (Fisher et al., 2024).

### Optimism Bias Model

We formalise optimism as a state factor in the generative model that can be applied across simulations and tasks. We use the state factor (**D**), learnt in our first simulation experiment, in the subsequent two tasks to represent how the agent may implement an optimism bias in a wide range of situations, where agents can use prior beliefs flexibly in different scenarios. Thus, applying the state factor (**D**) from one model to another allows us to investigate how one may use the same prior belief across task settings.

The optimism hidden state factor (**D**) encodes the high precision likelihood biased towards positive outcomes for each agent. This belief formalises the hypothesised role of optimism in everyday life, for example, ‘If I go to the party, I am likely to meet someone I connect with’ or ‘If I work out, I am likely to reduce my risk of cancer’ where the (optimistically overestimated) positive outcomes are meeting someone to connect with and reducing the risk of cancer, respectively. The optimism bias is the belief that the agent must be in an environment that affords positive outcomes. It is important to consider the relationship between optimism and the environment to understand when optimism may not be adaptive, such as environments that do not afford good outcomes. An agent may optimistically believe that if they go fishing, they will catch a fish, but if they live by a polluted river where all the fish have died, then optimism is not adaptive as the environment does not afford opportunities for a good outcome (see Fisher & Hohwy, 2024 for discussion of the relationship between optimism and the environment). This has practical implications for optimism as an intervention, as it is important to ensure that optimism is only increased for an individual if they are in an environment where there are good outcomes to obtain. Increasing optimism for those living in war zones or those who are experiencing homelessness, without removing them from that area or providing them with a home, would not be adaptive.

The optimism model presented here is an optimism hidden state factor (**D**) that has two states, so that the agent can be in an optimistic or pessimistic state. We refer to the optimism bias in terms of the probability of the agent being in either state. For example, if the optimism state factor for an agent is **D** = [0.8, 0.2], the agent would be at optimism level 0.8.

It is important to note that active inference as such implies what can be called a generic optimism bias, in the sense that agents always select policies under the belief that the policy that best minimises EFE will maximise their preferences (Parr et al., 2022). In this paper, we use optimism bias in a more specific sense to denote a precise biased belief that positive outcomes are more likely when conditioned upon an optimistic contextual belief. The former is a property of policy selection in general while the latter results from a conditionalization of the likelihood (**A** matrix) on the hidden state factor encoding the degree of optimism and pessimism of the agent. This formulation creates belief updating and policy selection that is not captured in a preference only model.

### Simulation Overview

**Figure 1:**
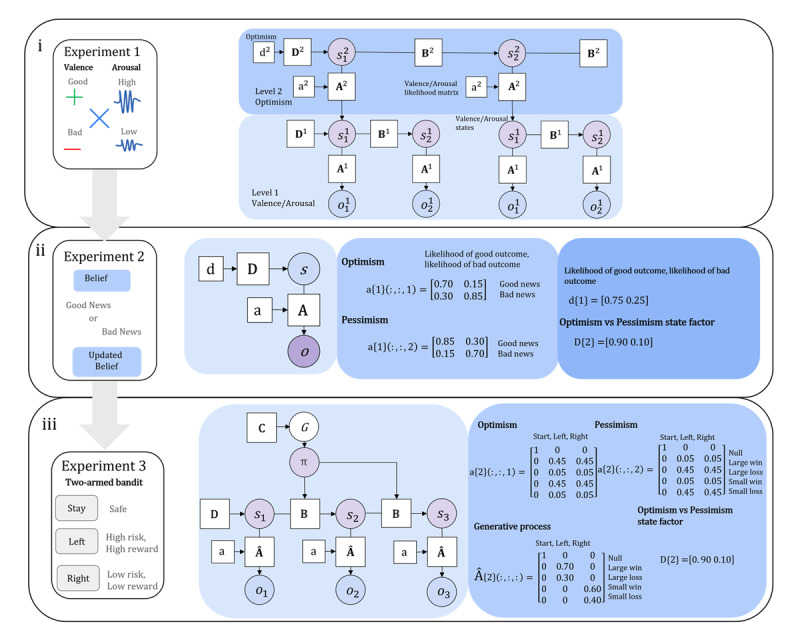
**i**. The left panel shows Experiment 1, which is a simulation where the agent is exposed to negative and positive valence and high and low arousal events. This simulates how the optimism bias is lost or maintained during development. The right panel shows a graphical depiction of the hierarchical active inference model. Level 1, observation level, sets the valence and arousal observations (*o*^1^) for the valence and arousal states (*s*^1^) that provide the observations for level 2. Level 2, state level, establishes the optimism bias, where the optimism bias is a state factor (**d**^2^) that is learnt over time. **A**^2^ is the likelihood mapping of optimism states to valence and arousal observations. **a**^2^ is the likelihood mapping the agent learns over time. **ii**. The left panel shows Experiment 2 which is the belief updating task where the agent updates their belief to good or bad news. The right side of the figure displays a simplified graphical depiction of the active inference model of the belief updating task. The centre panel shows the generative model likelihood matrix (**A**). The right panel shows the optimism state factor (**D**) and the state factor corresponding to the prior belief of a good or bad outcome (**d**). See main text for full explanation of the model **iii**. The left panel shows Experiment 3 which is a modified version of the two-armed bandit task where the agent can opt out of selecting an arm and stay safe. The right side shows a graphical depiction of the active inference of the two armed bandit task. The figure displays the likelihood matrices for the generative model that are conditioned on the optimism and pessimism state factor (**D**), as well as the likelihood matrix of the generative process (**Â**). See the main text for a full explanation of the model. See the MATLAB code for a view of all matrices and vectors in the model https://github.com/bethfisher-hub/optimism_simulations/.

In order to explore how optimism bias, formalised as a high precision likelihood biased towards positive outcomes, relates to development, belief updating, and action selection, we ran three simulation experiments (Figure 1). First, we investigate how optimism can be lost or maintained during (*in silico*) childhood. (See Smith and colleagues for an active inference model of synthetic “childhood” and “adulthood”. (Smith et al., 2019)). The optimism learnt from the first simulation is then applied to Experiment 2 and 3, with the aim of exploring how the same agent would perform in different environments. Experiments 1 and 2 involve minimising marginal free energy (*F*_*π,*_*_τ_*) and Experiment 3 involves minimising both *F*_*π,*_*_τ_* and expected free energy (*G*_*π*_).

### Experiment 1: Simulating Loss of Optimism Bias

Individuals with higher optimism have improved cardiovascular responses to stress, which leads to healthy arousal outcomes (Terrill et al., 2010). In contrast, individuals with anxiety can experience high arousal (Alkozei et al., 2015; Beidel et al., 1985; Rowa et al., 2017). To explore how optimism may be related to arousal we use a hierarchical active inference model. The model simulates how optimism bias can plausibly be lost during development in childhood. We simulate how children in environments that expose them to a greater number of high arousal and negative valence events, whether this be from a low socioeconomic environment, hostile child-rearing or other, cause them to lose an already existing optimism bias.

Our hierarchical active inference model has two levels (Figure 1i). The first level, the level of observations, exposes the agent to valence and arousal observations (*o*). This is representative of the life events a child may have that cause them to experience different valence and arousal states (*s*). Learning in the hierarchical model occurs at the second level, the state level, and there is no learning for the level of observations. The level of observations serves to create a set of arousal and valence states for the agent. There are no policies (*π*) at either level as the simulation aims to model how optimism is affected from exposure to events in childhood. Although children develop other behavioural responses as a result of exposure to negative and positive valenced events, such as avoidance strategies, the aim of the current model is not to simulate these behaviours.

The agent has two state factors (**D**^1^ vectors), which represent valence and arousal. The agent can be in a high or low arousal state and in a positive or negative valence state. A high arousal state is a state that causes higher physiological arousal measures such as heart rate and blood pressure, whereas low arousal states cause lower heart rate and blood pressure. Before the simulation, the agent begins with the belief it can be in all states equally. Positive valence states are states that cause emotions such as happiness, joy, and love, where negative valence states cause emotions such as sadness and anger. The arousal and valence states are representative of what different experiences may cause the child to feel. For example, a high arousal state and positive valence state might represent playing with other children in a park. A high arousal state and negative valence state could be being told off angrily by a parent.

There is a one-to-one mapping with the state factor and outcome (**A**^1^ matrix), that is, if the agent is in a high arousal state, it has a high arousal outcome. The transition probability (**B**^1^ matrix) is an identity matrix, which means the agent stays in the same state for each trial.

We define a healthy environment for an agent to develop in as one with more positively valenced high and low arousal states. In contrast, an agent that develops in a less healthy environment will be in more negatively valenced high and low arousal states.

The state level in the hierarchical active inference model encodes the agent’s optimism bias. We model optimism as one state factor, the agent can be in either an optimistic state or a pessimistic state. The agent begins with an optimistic state factor of [0.8, 0.2]. As optimism is a robust bias that is seen across species and also adaptive, we hypothesise that humans and animals alike are innately optimistic and therefore begin life with an optimism bias (Bateson et al., 2011; Iki & Adachi, 2023; Lagisz et al., 2020). In the state level of the model the agent changes its optimism or pessimism through exposure to high and low arousal and negatively and positively valenced events, where high valence observations are considered more likely conditional on the optimistic hidden state factor, and vice versa.

As the model is hierarchical, the posterior belief over states at the first level (i.e., the valence and arousal states) form the observations for the second level; this means the second level learns at a slower rate than the first level. For the valence state, if the agent is in an optimistic state then it believes that it is more likely to have a positive valence outcome, and if it is in a pessimistic state that it is more likely to have a negative valence outcome, the valence matrix (**a**^2^) is multiplied by 1000 so that new observations do not change the likelihood mapping, i.e, there is no learning. For the arousal outcomes, the agent begins with a flat likelihood mapping (**a**^2^) between high and low arousal observations and optimistic and pessimistic hidden states. The agent learns the likelihood mapping for arousal over time (**a**^2^). By learning the likelihood mapping for arousal, the model simulates how children exposed to repeated negative events can infer a pessimistic state from being exposed to repeated high arousal outcomes co-occurring with negative valence with greater frequency in biased environments; conversely, we simulate how children who are not exposed to such statistically biased environments do not develop this pessimistic response. Importantly, the likelihood for mapping arousal is not an expression of the optimism bias itself but rather relates the bias to a developmental context.

As the agent receives valence and arousal observations across its *in silico* childhood, it changes its belief about what state it is in. If an agent observes more negative valence events across low and high arousal observations, it receives more information that it is in the pessimistic state. This then changes the belief of the agent, so that the agent begins to believe it is in a pessimistic state. The state transition matrix (**B**) is an identity matrix and the agent stays in their optimistic or pessimistic state across that set of trials. There are no policies or preferences. The agent is learning from their observations but cannot act to change these observations.

We simulate 200 agents where the arousal and valence states are randomly selected and ordered. The observation level is 52 trials which is representative of the 52 weeks in a year. And the state level is 5 trials which is representative of the first 5 years.

### Experiment 2: Optimism Bias Belief Updating Task

One method of measuring optimism is with Sharot and colleagues’ belief updating task (Bottemanne et al., 2022; Garrett et al., 2014; Sharot et al., 2007). In this task, participants are asked to estimate their risk of a bad event, for example chance of getting cancer, then they are told their estimate is either higher or lower than their actual risk. Good news is when their risk is lower than their estimate and bad news is when their risk is higher than their estimate. Optimism is measured by how much the participant updates their belief to good versus bad news, where optimists update more to good news than bad news. The asymmetry in belief updating is how the optimism bias is maintained – when individuals come up against negative evidence against their beliefs, they update their beliefs less (Sharot, 2011). We next simulate the belief updating task to ground the model in this experimental literature on optimism bias, which allows us to test whether the formulation of optimism as a high precision likelihood biased towards positive outcomes reproduces key empirical findings.

For the belief updating task there are two hidden state factors. The first hidden state factor corresponds to the prior belief that the agent has about the likelihood of a good or bad outcome for the life event in that trial. For example, the trial could be the belief that the agent will not get cancer (likelihood of good outcome) or get cancer (likelihood of bad outcome). The second hidden state factor is the optimism bias state factor which controls how much the belief is updated to good versus bad news. The hidden state factor generative process is separate from the generative model as the agent updates their belief in response to a good news outcome or a bad news outcome. The first hidden state factor, the belief about the likelihood of a good or a bad outcome, is equipped with a conjugate Dirichlet prior so it can be learned after the trial (this is how the belief updating is calculated). In contrast, the second hidden state factor, the optimism bias state factor, has a very precise conjugate prior so that no learning occurs. We use the optimism state factor learnt in the Experiment 1 simulation (Figure 1ii).

The task has two outcomes: good news and bad news. There is one likelihood matrix (**A**) and the likelihood generative process is separate from the generative model. In the generative process, the likelihood matrix (**Â**) is an identity matrix as the good versus bad news outcome is the same regardless of the optimism of the agent. For the generative model (**a**), if an agent is in an optimistic state they have a higher precision likelihood biased towards positive outcomes, whereas for the pessimistic state they have a higher precision likelihood biased towards negative outcomes.

Each trial in the belief updating task is represented in one model. The update to good versus bad news is calculated by the difference in the initial hidden state factor belief (**d**) likelihood of a good outcome and likelihood of a bad outcome and the hidden state factor after the observation (***d***_*trial*__+1_).

\[Good\,\,news\,\,update = \,{d_{trial}}\,[1,1] - {d_{trial + 1}}\,[1,1]\]

\[Bad\,\,news\,\,update = \,{d_{trial\,}}[2,1] - {d_{trial + 1}}\,[2,1]\]

To calculate the belief updating for good news, the belief updating average is taken from good news trials where the agent receives a good news outcome. To calculate the belief updating for bad news, the belief updating average is taken from bad news trials where the agent receives a bad news outcome. We run the model 70 times for 70 trials. The prior beliefs of likelihoods of good outcomes and likelihoods of bad outcomes are randomly generated.

\[Average\,good\,news\,update,\,\,where\,N\,is\,number\,of\,trials\,\, = \,\,\frac{{\mathop \sum \nolimits_{i = 0}^n Good\,news\,updates}}{N}\,\,100\]

To determine what A matrix precision best replicates the optimism bias literature results, where the A matrix precision sets how much an agent updates to good vs bad news depending on whether they are in an optimistic or pessimistic state, we swept the A matrix precision parameters (*a*_*good*_, *a*_*bad*_). This is where we simulated different values in the A matrix rather than hard coding a pre-defined value, to determine what best replicated previous findings. The A matrix precision parameters for the optimistic state factor are shown below. The final parameters selected for the optimistic and pessimistic states are in Figure 1ii.

\[a\left\{ 1 \right\}\left({:,\,\,:,1} \right) = \,\left[ {\begin{array}{*{20}{c}}
{{a_{good}}}&{\left({1 - {a_{bad}}} \right)}\\
{(1 - {a_{good}})}&{{a_{bad}}}
\end{array}} \right]\]

### Experiment 3: Performance on a two-armed bandit task with various levels of the optimism bias

Finally, to explore the influence of optimism on actions, we leverage a two-armed bandit task with the same optimism hidden state factor from Experiment 1 and 2 (Figure 1iii). For the two-armed bandit task there is a state factor (**D**) corresponding to the location of the agent in the task. The agent can be positioned at the start of the task, or at the left arm, or the right arm. There is one outcome modality (**Â**) encoding the location of the agent, they observe either being at the start, in the left arm, or right arm. As the agent always knows the location they are in, the location likelihood (**A**) is an identity matrix.

In the task, the agent can receive a null outcome, a large win, a large loss, a small win, or a small loss. The reward outcome modality (**Â**) sets the probability of the wins and losses in each arm. For the generative process, the reward outcomes are the same regardless of the optimism of the agent as this sets the outcomes for arms in the task. We separate the generative model from the generative process to show how optimism changes the beliefs about the outcomes of the task. In the generative model, a higher precision likelihood biased towards positive outcomes (**a**) is conditioned on the optimism of the agent. In this task, the agent has a preference for wins. The optimistic agent has a higher precision likelihood biased towards wins compared to losses and null outcomes.

The agent can choose from three policies (*π*); select the left arm, select the right arm, or remain at the start. If the agent selects the right or left arm they have a chance of winning money but they also have a chance of losing money, so there is risk involved in that decision. If the agent decides to remain at the start they will not win any additional money, but they will not lose the money they have. There is no risk remaining in the start position, but there is also no chance of reward. For the preferences (**C**), the agent has the highest preference for a large win, and the lowest preference for a large loss. In the middle of these preferences is a null outcome, where although the agent would rather observe a win to null it would rather observe a null to a loss. We set there to be no preference for the null outcome as this does not change the overall position of the agent (since they do not gain or lose anything). The preferences are the same for all agents and are not updated throughout the experiment, that is, there is no **C** matrix learning.

The left arm results in large win and large loss outcomes, representing an action where there is a lot to gain but also more to lose. This could be going to a party where the agent does not know anyone, they could make new friends but there is a chance they awkwardly fail to meet anyone. The right arm results in small win and small loss outcomes. In the world, this is representative of a scenario that is still more risky than doing nothing but not as risky as the other option. This could be going to the party that friends have put on, where there is a small chance they will not have a good time but overall it should be a pleasant experience with known friends albeit falling short of meeting someone entirely new. The action to not participate in that trial is equivalent to staying at home and not going to any party. There is no chance of meeting anyone new, but nothing bad will happen either. One will remain in the same position.

Each agent completed 60 trials. We summed the overall performance: the large win is gaining $4, the large loss is losing $4, the small win is gaining $1 and the small loss is losing $1.

## Results

### Loss of Optimism Bias

**Figure 2:**
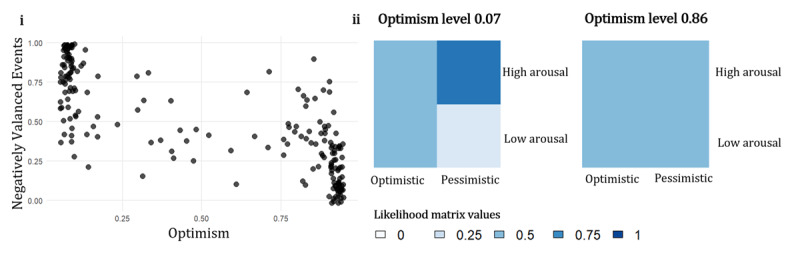
**i**. Resulting optimism state factor level versus the proportion of negatively valenced events the agent was exposed to. **ii**. Two examples of resulting likelihood matrices from the simulation for an agent with learned optimism level 0.07 and an agent with learned optimism level 0.86 at the end of the simulation.

The simulation explores how optimism may be lost in development from exposure to negatively valenced events. In Figure 2i, we plot the number of negatively valenced events and the optimism level learnt at the end of the simulation. The optimism level is normalised accumulated Dirichlet counts associated with the optimism state factor at the end of the simulation, which is the belief that the agent is in an optimistic state. For the negative valenced events, we take the proportion of these events the agent experiences across the time. The results show that the more negatively valenced events the agent is exposed to the lower level of optimism they have. The findings of the simulation reproduce empirical literature on the optimism bias as we show that the environment the agent is exposed to during development impacts their optimism (Heinonen et al., 2005, 2006). Additionally, we find that there is not a one-to-one mapping between the number of negatively valenced events and the optimism bias. This suggests that even in a simple, idealised model there can be variability in the loss of optimism bias during development which in the real world would likely be due to variability in the timing of exposure of negative events, and variance in genetic and protective factors.

Figure 2ii shows an example of two state level likelihood matrices that were learnt after the simulation concluded. The agent with optimism level 0.07 had more high arousal outcomes cooccurring with negative valence, leading it to infer a precise mapping between high arousal and a pessimistic state. The agent had a proportion of 0.98 negatively valenced events. This led to a set of beliefs plausibly describing an anxious phenotype with high arousal events leading the agent to infer a pessimistic state. As the agent was exposed to mainly negative events it learnt that the environment consists of negative experiences, and learns to expect these.

In contrast, the agent with optimism level 0.86 has a flat likelihood matrix at the end of the simulation, meaning in each state it can experience high and low arousal without being led to infer that it is in either an optimistic or pessimistic state. These arousal outcomes are consistent with a healthy agent. Even when the agent is in a pessimistic state it still maintains high and low arousal equally. Such an agent may enter a stressful situation and be in a pessimistic state but not experience a disproportionate amount of high arousal, which may be associated with a state of panic. This agent had a proportion of 0.56 positively valenced events during the simulation, it still experienced negatively valenced events but had positive experiences as well. This suggests that to maintain optimism and a healthy arousal response, the agent does need to experience some negative events. For all the agents, there was variability in the resulting likelihood matrix after the simulation, depending on the number of events and time or exposure. The variability in the likelihood matrix is similar to how we interpret the variability in the optimism, that is, a range of factors could contribute to an individual’s arousal response, resulting in variability.

### Performance on Belief Updating Task

**Figure 3:**
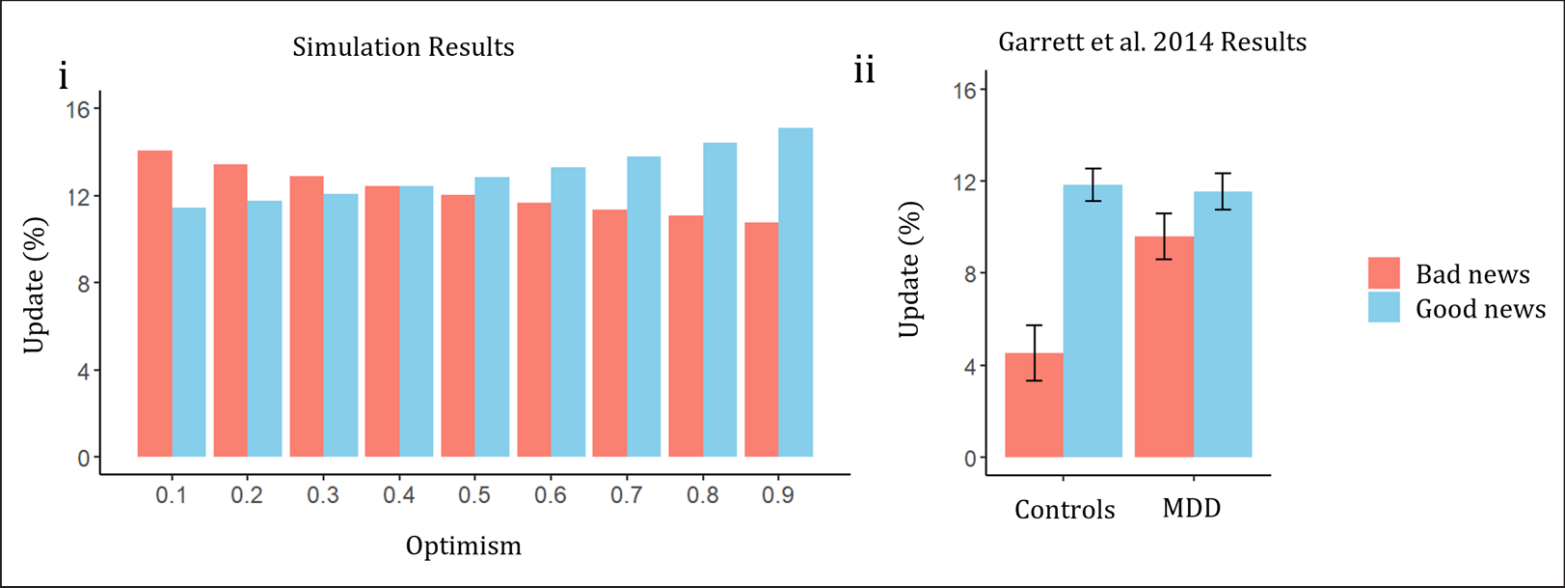
**i**. Mean percentage of updating beliefs to good versus bad news for each agent. The x axis plots the agent by its level of optimism. **ii**. Belief updating task results from Garrett et al. 2014 in cohort of healthy controls (n = 14) and patients diagnosed with major depressive disorder (MDD) (n = 15). Results are taken from Figure 2 in Garrett et al. 2014. Good news is plotted in blue and bad news is plotted in orange.

The results of the belief updating task showed that for increasingly optimistic agents, the agents updated their beliefs more to good news versus bad news. This result replicates findings in empirical research on optimism where optimists learn more from good versus bad news. Figure 3i shows the asymmetry in belief updating as a function of optimism. We find the highest asymmetry in belief updating was for agents with optimism of 0.9. As optimism decreased and pessimism increased, the agent updated more to bad news than good news. Figure 3ii plots the results from Garrett and colleagues (2014) to compare the simulated results to empirical data. Garrett and colleagues showed that participants with Major Depressive Disorder (MDD) update more to bad news than the controls. The controls in this study have results similar to our 0.9 optimism agent, implying, in line with our model, that people in the healthy population are optimistic. The MDD results are comparable to optimism level 0.5, which can be considered an example of depressive realism.

### Performance on Two-Armed Bandit Task

The two-armed bandit task explored how optimism related to actions of the agent. This modified two-armed bandit task was a novel task design so there were no empirical findings to compare the results. However, as optimism involves increased engagement in the world, we hypothesised that increased optimism levels would result in increased task engagement.

**Figure 4:**
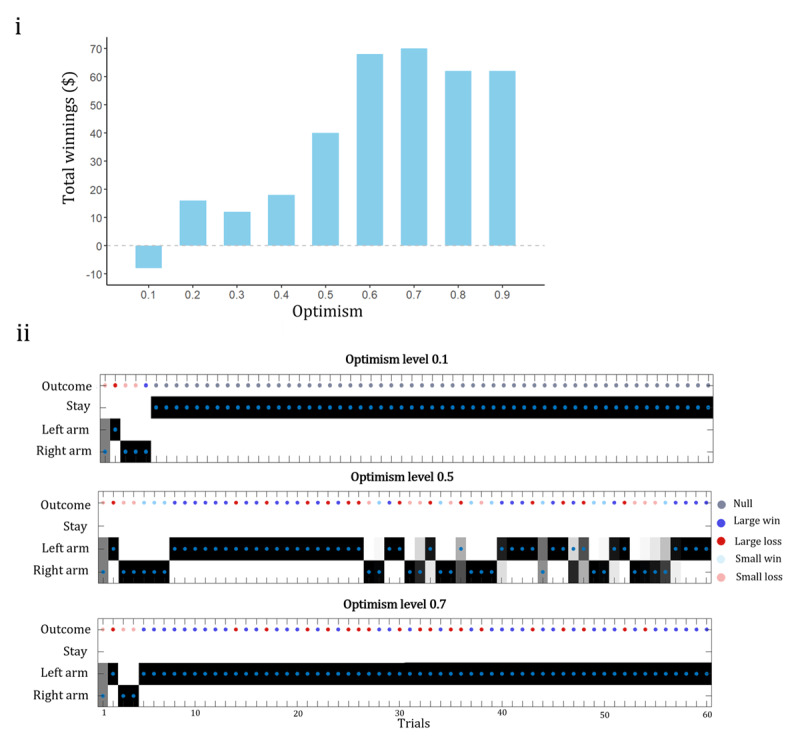
**i**. Total winnings on the two armed bandit task for each agent. The x-axis plots each agent as their level of optimism. **ii**. Action probabilities and chosen actions for three agents in the two-armed bandit task. The blue dots indicate the actions the agent took and the grey indicates the action probability of the agent. The outcome line plots the large win, large loss, small win, small loss and null outcomes of the action. Agents with level optimism 0.1, 0.7 and 0.5 are plotted.

We found that the performance on the two-armed bandit task varied depending on the agent’s level of optimism. Figure 4i plots the total winnings of the agent and their optimism level. The best performing agent had 0.7 as their level of optimism. Agents from level 0.1 – 0.4 had lower total winnings than the agents 0.5–0.9. To further highlight how the levels of optimism affected task performance we plot the actions and outcomes of the best performing agent (0.7) and the pessimistic agent (0.1) and an 0.5 agent (Figure 4ii).

Figure 4ii shows the agent who received the highest total winnings, agent with optimism level 0.7. After a few initial trials where the agent chose both arms, the agent stayed selecting the left arm. This strategy results in the highest wins overall as it is the high risk, high reward arm. The actions of the optimism level 0.7 agent show that the agent always engaged with the task and they never selected the action of stay. This finding translates to behaviours of optimists who engage in the world as they believe their actions will result in a good outcome.

In contrast, the most pessimistic agent, optimism level 0.1, selected the left and right arm for the first few trials and then decides to select the stay action for the remaining trials. This series of actions resulted in a net loss for the pessimistic agent. This behaviour is what we would expect from an individual with depression, they do not believe they will achieve their preferred outcome in the world so engage less than an optimistic individual. The action of stay is a safe decision as there is no risk of any loss. If an agent does not believe they would achieve a good outcome, the decision to stay protects them from experiencing loss.

Figure 4ii also plots the actions of the 0.5 optimism level agent. This agent selects both left and right arms throughout the trials. When receiving losses in one arm it switches to the other arm. The agent does not experience enough losses to decide to select the stay action. Appendix 1 includes plots for the raw number of wins and losses; these plots illustrate how one could generate predictions of outcomes that are close to experimentally quantifiable behavior.

## Discussion

In this paper, we have developed an active inference model of optimism bias. The model we have presented is domain general, meaning it can be applied to different task settings and environments by running tasks that test optimistic belief updating and optimistic action with a shared optimism hidden state factor. Using our model, we have explored how the optimism bias could be lost during development, replicated key findings in the optimism literature showing asymmetric belief updating to good and bad news, and showed how such beliefs change action through a modified version of the two-armed bandit task. The simulation findings provide a computational proof-of-concept for the active inference model of optimism bias. This lays the groundwork for future research on how we can measure the optimism bias in human participants, with the aim of using manipulations of optimism as a mental health intervention.

In Experiment 1, the hierarchical active inference model demonstrated how the optimism bias could plausibly be lost during development. The results show that exposure to more negatively valenced events results in less optimism bias, in line with empirical work showing that children exposed to more negative valenced events are less optimistic (Heinonen et al., 2005, 2006), a finding for which we provide a plausible computational basis. Environmental risk factors such as socioeconomic status in childhood (Heinonen et al., 2006) and hostile child rearing attitudes in mothers have also been found to have lower optimism levels later in life (Heinonen et al., 2005).

The relationship between the optimism bias and exposure to negatively valenced events was variable, as there was not a one-to-one mapping of the number of negatively valenced events and the level of optimism. The variability in results can be due to the timing of exposure to the events; thus, in active inference, when learning about the environment, observations that happen earlier (when Dirichlet concentration parameters are small) have more weight. For example, if an agent initially experiences a positively valenced event and then a negatively valenced event, the negatively valenced event will affect them less. This variability may be shown in the literature where different experiences that would be associated with positive valence result in protective factors for well-being for children (Buchanan et al., 2002; Moore & Ramirez, 2016). More research is needed to explore in the model when and how the timing of exposure effects might occur; this could lead to empirical predictions of how quickly optimism can be lost, and how slowly it can be regained. There may also be a genetic component that contributes to the variability in optimism as it has been suggested that additive genetic factors account for about a third of the variation in optimism levels (Bates, 2015; Mosing et al., 2009).

For the likelihood matrix results in the simulations in Experiment 1, we are asking: “how often does the agent experience a high arousal outcome if they are in an optimistic or pessimistic state?” (where the higher the agent’s level of optimism, the more often they will be in an optimistic state). The results indicate that an agent who was exposed to more negatively valenced events and had lower optimism also had a likelihood matrix that resulted in more high arousal outcomes. Individuals with anxiety can experience more high arousal states (Alkozei et al., 2015; Beidel et al., 1985; Rowa et al., 2017), and this sign of anxiety is plausibly shown in the likelihood matrix results for some low optimism agents. In contrast, for a healthy agent, they should maintain their arousal equally in either a pessimistic or optimistic state. This is because maintaining arousal means the agent does not get overly anxious or stressed, where anxiety and stress are related to high arousal. Therefore, for the likelihood matrix, we should be able to observe that less high arousal outcomes should be observed for optimistic states for agents with a higher optimism level. The results indicate that agents who were exposed to more positive valenced events with higher optimism had a flat likelihood matrix. This finding is consistent with experimental results in the literature that find optimism is associated with healthier arousal responses to stress (Birkeland et al., 2017; Brydon et al., 2009; Oveis et al., 2009). Additionally, maintaining arousal in these states is a key concept behind meditation or breathwork interventions for anxiety and stress (Pascoe et al., 2017).

The likelihood matrices showed variability due to the timing of the negative or positive events. It has been suggested that valence and arousal can be highly variable depending on the individual, and the variability in the model is a strength in this regard, as it can capture this feature (Kuppens et al., 2013). Experiment 1 builds on the literature of emotional concept learning in development in active inference, providing further examples of how one can model *in silico* childhoods (Smith et al., 2019). The Experiment 1 results show how our active inference model of optimism bias can link to the literature on development in children, which supports our modelling approach and could be used to generate quantitative predictions and potentially be explored for phenotyping in computational psychiatry.

In Experiment 2, the optimism bias state factor from Experiment 1 was simulated in the belief updating task. The belief updating task results showed that with higher levels of optimism, agents updated their beliefs more to good rather than bad news. The asymmetry in belief updating shown in the simulation is similar to the asymmetry in belief updating found in the literature (Garrett et al., 2014; Korn et al., 2014; Sharot, 2011). Additionally, the task found that agents who are in a pessimistic state update more to bad news rather than good news, replicating the behavioural phenotype of people with MDD (Garrett et al., 2014). Although similar, the results of Experiment 2 are not the exact same as the belief updating in Garrett and colleagues; such differences are not unexpected as the computational model is an abstraction of the process of completing the task (Garrett et al., 2014). Creating a model that precisely replicates the findings of empirical results may require a more complex model with other processes contributing to the participant’s decision making, or hard coding the model. The model here was created from a theoretical basis to test the dynamics of the optimism bias, and using a more complex model or hard coding would not be in line with this aim.

Optimism bias is typically assessed by the belief updating task used in Experiment 2, however this does not capture optimistic action. As the adaptive aspect of the optimism bias is mediated by increased engagement with the world through actions, it is beneficial to be able to measure and model that action. We therefore developed a modified two-armed bandit task that tests optimistic action, in Experiment 3. The main advantage of the task is that the agent can choose to not select the left or right arm, selecting to stay instead, which is the action of not engaging in the task; here choosing to stay is similar to deciding to stay at home and not go to the party or not going out to exercise. Importantly, by selecting to stay the agent does not lose anything, as they remain in the same position. It is not a negative action, but a safe one where potential rewards by engaging in the environment are missed (cf. lost opportunities).

The results of Experiment 3 show what level of optimism is best for maximum reward in this task setting. Specifically, optimism level 0.7 results in the highest amount of winnings, and when considering the action selection, this agent selected the left arm, which is the high risk high reward arm that overall will result in the most winnings. Hence, the optimistic agent is not deterred by losses and can keep engaging with the task even after a few losses in a row. This action selection can be conceived in terms of how an individual with the optimism bias would engage in the world – even if they come up against some losses they will keep engaging (e.g., an optimist might go to a party and have a bad experience, but be able to attend another party the next week). In contrast, the actions of an agent with the lowest level of optimism, 0.1 resulted in an overall loss. The 0.1 level agent selects the left and right arm initially but after experiencing enough losses they decide to select the action of stay for the remainder of the trials. These actions are similar to what may be observed with someone who lacks the optimism bias, when experiencing any losses they decide to stay safe even if this means there is no opportunity for a reward (e.g., someone who lacks the optimism bias may go to the party, have a bad time, and decide not to go again). This result may speak to individuals with depression, who decide to stay at home, often suffering with apathy.

Interestingly, in the two-armed bandit task, the overly optimistic agent is less optimal, beginning to experience more losses. This finding is consistent with empirical research suggesting that in certain scenarios optimism can be related to negative consequences. For optimism and immune response, it has been shown that optimism helps to maintain T cell percentages and natural killer cytotoxicity cells (NKCC) when exposed to a stressor (Cohen et al., 1999), where the stressor in this study was any life event the participant found stressful. However, when the stressor lasted more than one week, optimism was shown to be associated with a lower T cell percentage and NKCC compared to pessimism. It is suggested that as optimistic individuals engage more with a stressor, they may cope well for a small stressor that they can resolve and overcome, resulting in a positive immune response, whereas when the stressor is larger, they have the same engagement without the ability to overcome, resulting in more stress and a weakened immune response. In contrast, individuals who lack optimism do not engage with either type of stressors. This results in a negative outcome for the mild stressor as they miss the opportunity to overcome it when they could. Yet ignoring the large stressor can be a positive outcome as the individual is not constantly thinking and worrying about this issue. These are important considerations for developing an optimism bias intervention, as there may be circumstances where optimism is suboptimal.

In the two-armed bandit task, the agents were in an environment that afforded them with positive outcomes, so, since they had opportunities to win, it was adaptive to be optimistic. The optimism bias model presented here modelled the agent’s belief in their actions without their beliefs about the world they were in, however for a clinical intervention one would need to also take into account the environment the patient was in. In particular, increasing optimism for an individual in an environment that does not afford any positive outcomes would not be adaptive. For example, increasing optimism for someone experiencing homelessness who are not in an environment with opportunities for a home would not help the individual. Thus, the beliefs the agent has about their actions resulting in positive outcomes must be coupled with the world they are in, which can afford positive outcomes (Fisher & Hohwy, 2024).

The active inference model of the optimism bias presented here is domain general, meaning it applies to more than one task setting. Modelling optimism as a state factor that can be used in different tasks demonstrates how an individual may have a bias that affects different aspects of their decision making in many contexts. As shown in the three experiments, we provide evidence for a domain general model by replicating findings and behaviours of optimistic individuals in various contexts. This has clinical relevance, as one could measure an individual’s optimism in different settings to gain more information on whether an optimism intervention is best suited for that individual. These advantages are one of the aims of the field of computational psychiatry (Adams et al., 2015; Browning et al., 2023; Huys et al., 2016; Montague et al., 2012).

One limitation of the current study is that the model presented here has not been fitted to data. Simulations provide us with some insight into possible mechanisms of processes such as optimism but fitting the models to data is required to more fully clarify the empirical underpinnings of the model. Another potential limitation concerns the clinical relevance of the model; even if the model does fit to participant data there will need to be further work addressing if the model is able to find meaningful parameter differences for healthy individuals and psychiatric disorders. This is not a limitation of only this model but a challenge for the field of computational psychiatry (Karvelis, Paulus & Diaconescu, 2023).

The future directions of this work include collecting and fitting participant data from healthy and depressed cohorts. The design of the simulations are such that the optimism bias model can be tested for its validity for use in clinical settings. Future work includes testing the optimism bias model levels for depressed cohorts to determine if the model successfully finds depressed individuals have lower optimism levels. For depressed cohorts, the modified two-armed bandit may provide a novel way of testing those who may benefit from a behavioural activation treatment (Soucy Chartier & Provencher, 2013; Tindall et al., 2017), by understanding those who chose not to engage in the task. Additionally, using the optimism model and modified two-armed bandit task, we can begin to explore the relationship between optimism and behavioural activation, and if treatments that target behavioural activation could use an optimism intervention. Longitudinal studies over development could investigate how arousal related outcomes change in response to stressors whilst measuring optimism levels as found in Experiment 1. Other future work should investigate what treatments may increase the optimism bias and how these treatments can be computationally modelled using the optimism bias model.

## Conclusion

Here, we present and provide support for a domain general active inference model of the optimism bias. Using three simulations, we show how the optimism bias model applies to optimistic belief updating and optimistic action, relating this to the optimism bias in development. Our simulation results replicate findings in the literature and lay the groundwork for future work on the optimism bias model for use in a clinical setting.

## Figures and Tables

**Figure A.1:**
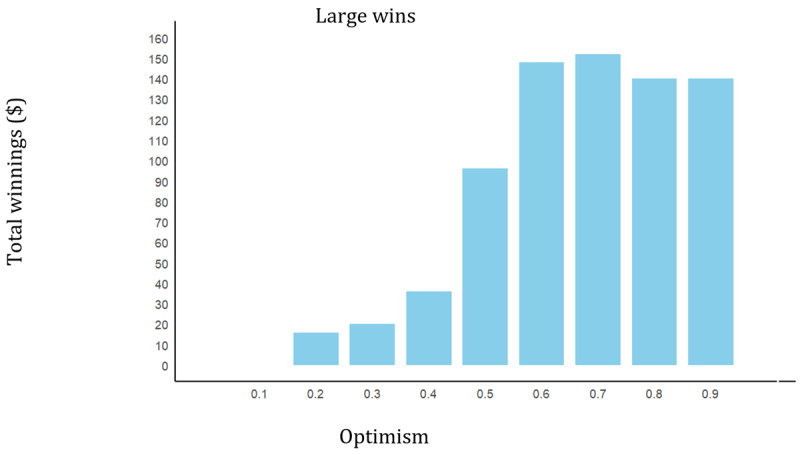
Total winnings from large wins on the two armed bandit task for each agent. The x-axis plots each agent as their level of optimism.

**Figure A.2:**
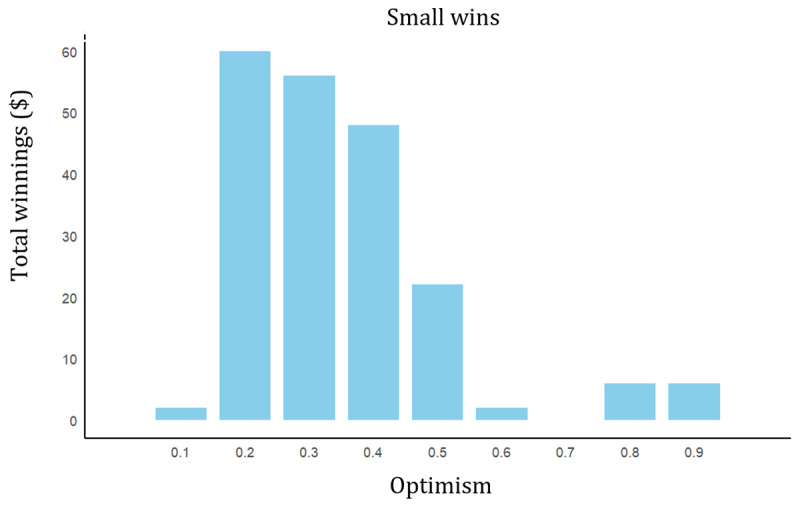
Total winnings from small wins on the two armed bandit task for each agent. The x-axis plots each agent as their level of optimism.

**Figure A.3:**
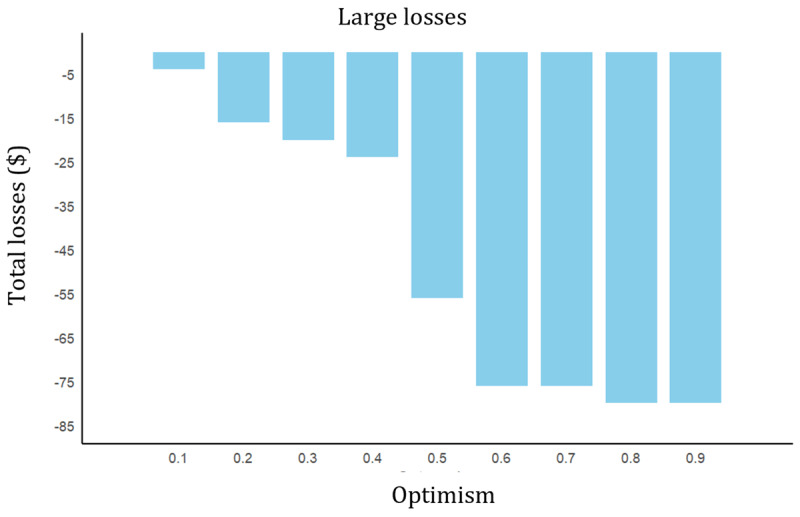
Total losses from large losses on the two armed bandit task for each agent. The x-axis plots each agent as their level of optimism.

**Figure A.4:**
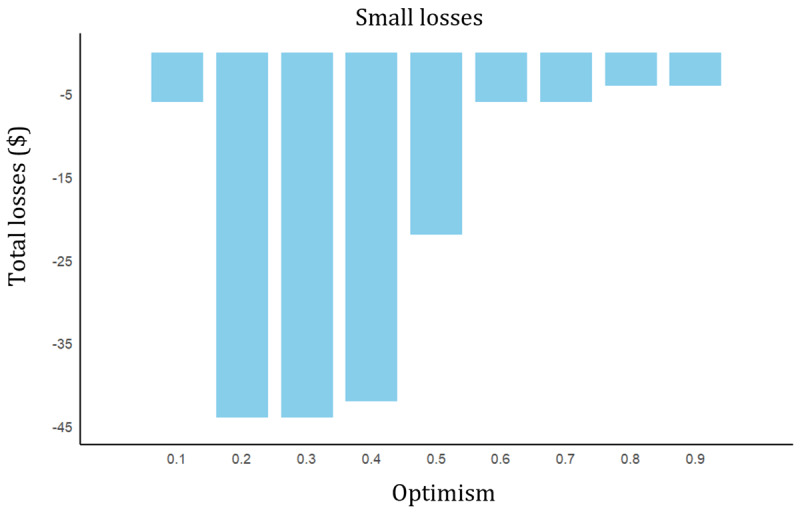
Total losses from small losses on the two armed bandit task for each agent. The x-axis plots each agent as their level of optimism.

## Software Note

Code used presented in this paper is openly available on the Github repository (https://github.com/bethfisher-hub/optimism_simulations/). Models were simulated in MATLAB (v.9.12.0, (R2022a) using the SPM academic software (http://www.fil.ion.ucl.ac.uk/spm/).

## References

[1] Adams, R. A., Huys, Q. J. M., & Roiser, J. P. (2015). Computational Psychiatry: Towards a mathematically informed understanding of mental illness. Journal of Neurology, Neurosurgery & Psychiatry, jnnp-2015-310737. DOI: 10.1136/jnnp-2015-310737 PMC4717449 26157034

[2] Alkozei, A., Creswell, C., Cooper, P. J., & Allen, J. J. B. (2015). Autonomic arousal in childhood anxiety disorders: Associations with state anxiety and social anxiety disorder. Journal of Affective Disorders, 175, 25–33. 10.1016/j.jad.2014.11.056 25590763 PMC4366038

[3] Bates, T. C. (2015). The glass is half full and half empty: A population-representative twin study testing if optimism and pessimism are distinct systems. The Journal of Positive Psychology, 10(6), 533–542. 10.1080/17439760.2015.1015155 26561494 PMC4637169

[4] Bateson, M., Desire, S., Gartside, S. E., & Wright, G. A. (2011). Agitated Honeybees Exhibit Pessimistic Cognitive Biases. Current Biology, 21(12), 1070–1073. 10.1016/j.cub.2011.05.017 21636277 PMC3158593

[5] Beidel, D. C., Turner, S. M., & Dancu, C. V. (1985). Physiological, cognitive and behavioral aspects of social anxiety. Behaviour Research and Therapy, 23(2), 109–117. 10.1016/0005-7967(85)90019-1 4004691

[6] Birkeland, M. S., Blix, I., Solberg, Ø., & Heir, T. (2017). Does optimism act as a buffer against posttraumatic stress over time? A longitudinal study of the protective role of optimism after the 2011 Oslo bombing. Psychological Trauma: Theory, Research, Practice, and Policy, 9(2), 207–213. 10.1037/tra0000188 27642805

[7] Bottemanne, H., Morlaas, O., Claret, A., Sharot, T., Fossati, P., & Schmidt, L. (2022). Evaluation of Early Ketamine Effects on Belief-Updating Biases in Patients With Treatment-Resistant Depression. JAMA Psychiatry, 79(11), 1124–1132. 10.1001/jamapsychiatry.2022.2996 36169969 PMC9520441

[8] Browning, M., Paulus, M., & Huys, Q. J. M. (2023). What is Computational Psychiatry Good For? Biological Psychiatry, 93(8), 658–660. 10.1016/j.biopsych.2022.08.030 36244802

[9] Brydon, L., Walker, C., Wawrzyniak, A. J., Chart, H., & Steptoe, A. (2009). Dispositional optimism and stress-induced changes in immunity and negative mood. Brain, Behavior, and Immunity, 23(6), 810–816. 10.1016/j.bbi.2009.02.018 19272441 PMC2715885

[10] Buchanan, A., Flouri, E., & Ten Brinke, J. (2002). Emotional and Behavioural Problems in Childhood and Distress in Adult Life: Risk and Protective Factors. Australian & New Zealand Journal of Psychiatry, 36(4), 521–527. 10.1046/j.1440-1614.2002.01048.x 12169153

[11] Cohen, F., Kearney, K. A., Zegans, L. S., Kemeny, M. E., Neuhaus, J. M., & Stites, D. P. (1999). Differential Immune System Changes with Acute and Persistent Stress for Optimists vs Pessimists. Brain, Behavior, and Immunity, 13(2), 155–174. 10.1006/brbi.1998.0531 10373279

[12] Conversano, C., Rotondo, A., Lensi, E., Della Vista, O., Arpone, F., & Reda, M. A. (2010). Optimism and Its Impact on Mental and Physical Well-Being. Clinical Practice and Epidemiology in Mental Health : CP & EMH, 6, 25–29. 10.2174/1745017901006010025 20592964 PMC2894461

[13] Cooper, J. A., Arulpragasam, A. R., & Treadway, M. T. (2018). Anhedonia in depression: Biological mechanisms and computational models. Current Opinion in Behavioral Sciences, 22, 128–135. 10.1016/j.cobeha.2018.01.024 29503842 PMC5828520

[14] Curzytek, K., Kubera, M., Trojan, E., Wójcik, K., Basta-Kaim, A., Detka, J., Maes, M., & Rygula, R. (2018). The effects of pessimism on cell-mediated immunity in rats. Progress in Neuro-Psychopharmacology and Biological Psychiatry, 80, 295–303. 10.1016/j.pnpbp.2017.04.034 28595946

[15] de Ridder, D., Fournier, M., & Bensing, J. (2004). Does optimism affect symptom report in chronic disease?: What are its consequences for self-care behaviour and physical functioning? Journal of Psychosomatic Research, 56(3), 341–350. 10.1016/S0022-3999(03)00034-5 15046972

[16] Fisher, E. L., & Hohwy, J. (2024). The Universal Optimism of the Self-Evidencing Mind. Entropy, 26(6), Article 6. 10.3390/e26060518 PMC11202793 38920527

[17] Fisher, E. L., Smith, R., Corcoran, A. W., Milton, L. K., Conn, K., Hohwy, J., & Foldi, C. J. (2024). Psilocybin increases optimistic engagement over time: Computational modelling of behavior in rats (p. 2024.05.16.594614). bioRxiv. 10.1101/2024.05.16.594614 PMC11442808 39349428

[18] Friston, K. (2022). Computational psychiatry: From synapses to sentience. Molecular Psychiatry. 10.1038/s41380-022-01743-z PMC7614021 36056173

[19] Friston, K. J., & Stephan, K. E. (2007). Free-energy and the brain. Synthese, 159(3), 417–458. 10.1007/s11229-007-9237-y 19325932 PMC2660582

[20] Garrett, N., Sharot, T., Faulkner, P., Korn, C. W., Roiser, J. P., & Dolan, R. J. (2014). Losing the rose tinted glasses: Neural substrates of unbiased belief updating in depression. Frontiers in Human Neuroscience, 8. https://www.frontiersin.org/article/10.3389/fnhum.2014.00639. 10.3389/fnhum.2014.00639 PMC4147849 25221492

[21] Goldway, N., Eldar, E., Shoval, G., & Hartley, C. A. (2023). Computational Mechanisms of Addiction and Anxiety: A Developmental Perspective. Biological Psychiatry, 93(8), 739–750. 10.1016/j.biopsych.2023.02.004 36775050 PMC10038924

[22] Heinonen, K., Räikkönen, K., & Keltikangas-Järvinen, L. (2005). Dispositional optimism: Development over 21 years from the perspectives of perceived temperament and mothering. Personality and Individual Differences, 38(2), 425–435. 10.1016/j.paid.2004.04.020

[23] Heinonen, K., Räikkönen, K., Matthews, K. A., Scheier, M. F., Raitakari, O. T., Pulkki, L., & Keltikangas-Järvinen, L. (2006). Socioeconomic Status in Childhood and Adulthood: Associations With Dispositional Optimism and Pessimism Over a 21-Year Follow-Up. Journal of Personality, 74(4), 1111–1126. 10.1111/j.1467-6494.2006.00404.x 16787430

[24] Hobbs, C., Vozarova, P., Sabharwal, A., Shah, P., & Button, K. (2022). Is depression associated with reduced optimistic belief updating? Royal Society Open Science, 9(2), 190814. 10.1098/rsos.190814 35127107 PMC8808098

[25] Houthooft, R., Chen, X., Duan, Y., Schulman, J., De Turck, F., & Abbeel, P. (2017). VIME: Variational Information Maximizing Exploration (arXiv:1605.09674). arXiv. 10.48550/arXiv.1605.09674

[26] Huys, Q. J. M., Maia, T. V., & Frank, M. J. (2016). Computational psychiatry as a bridge from neuroscience to clinical applications. Nature Neuroscience, 19(3), Article 3. 10.1038/nn.4238 PMC5443409 26906507

[27] Iki, S., & Adachi, I. (2023). Fearful snake pictures make monkeys pessimistic. iScience, 26(9), 107622. 10.1016/j.isci.2023.107622 37664603 PMC10474457

[28] Karvelis, P., Paulus, M. P., & Diaconescu, A. O. (2023). Individual differences in computational psychiatry: A review of current challenges. Neuroscience & Biobehavioral Reviews, 148, 105137. 10.1016/j.neubiorev.2023.105137 36940888

[29] Korn, C. W., Sharot, T., Walter, H., Heekeren, H. R., & Dolan, R. J. (2014). Depression is related to an absence of optimistically biased belief updating about future life events. Psychological Medicine, 44(3), 579–592. 10.1017/S0033291713001074 23672737 PMC3880066

[30] Krittanawong, C., Maitra, N. S., Hassan Virk, H. U., Fogg, S., Wang, Z., Kaplin, S., Gritsch, D., Storch, E. A., Tobler, P. N., Charney, D. S., & Levine, G. N. (2022). Association of Optimism with Cardiovascular Events and All-Cause Mortality: Systematic Review and Meta-Analysis. The American Journal of Medicine, 135(7), 856–863.e2. 10.1016/j.amjmed.2021.12.023 35123934

[31] Kube, T., & Rozenkrantz, L. (2021). When Beliefs Face Reality: An Integrative Review of Belief Updating in Mental Health and Illness. Perspectives on Psychological Science, 16(2), 247–274. 10.1177/1745691620931496 32818386

[32] Kuppens, P., Tuerlinckx, F., Russell, J. A., & Barrett, L. F. (2013). The relation between valence and arousal in subjective experience. Psychological Bulletin, 139(4), 917–940. 10.1037/a0030811 23231533

[33] Lagisz, M., Zidar, J., Nakagawa, S., Neville, V., Sorato, E., Paul, E. S., Bateson, M., Mendl, M., & Løvlie, H. (2020). Optimism, pessimism and judgement bias in animals: A systematic review and meta-analysis. Neuroscience & Biobehavioral Reviews, 118, 3–17. 10.1016/j.neubiorev.2020.07.012 32682742

[34] Lee, E., Jayasinghe, N., Swenson, C., & Dams-O’Connor, K. (2019). Dispositional optimism and cognitive functioning following traumatic brain injury. Brain Injury, 33(8), 985–990. 10.1080/02699052.2019.1606448 31055941

[35] Littman, M. L. (2009). A tutorial on partially observable Markov decision processes. Journal of Mathematical Psychology, 53(3), 119–125. 10.1016/j.jmp.2009.01.005

[36] Montague, P. R., Dolan, R. J., Friston, K. J., & Dayan, P. (2012). Computational psychiatry. Trends in Cognitive Sciences, 16(1), 72–80. 10.1016/j.tics.2011.11.018 22177032 PMC3556822

[37] Moore, K. A., & Ramirez, N. A. (2016). Adverse Childhood Experience and Adolescent Well-being: Do Protective Factors Matter? Child Indicators Research, 9(2), 299–316. 10.1007/s12187-015-9324-4

[38] Mosing, M. A., Zietsch, B. P., Shekar, S. N., Wright, M. J., & Martin, N. G. (2009). Genetic and Environmental Influences on Optimism and its Relationship to Mental and Self-Rated Health: A Study of Aging Twins. Behavior Genetics, 39(6), 597–604. 10.1007/s10519-009-9287-7 19618259

[39] Oveis, C., Cohen, A., Gruber, J., Shiota, M., Haidt, J., & Keltner, D. (2009). Resting Respiratory Sinus Arrhythmia Is Associated With Tonic Positive Emotionality. Emotion (Washington, D.C.), 9, 265–270. 10.1037/a0015383 19348538

[40] Parr, T., Pezzulo, G., & Friston, K. J. (2022). Active inference: The free energy principle in mind, brain, and behavior. The MIT Press. 10.7551/mitpress/12441.001.0001

[41] Pascoe, M. C., Thompson, D. R., Jenkins, Z. M., & Ski, C. F. (2017). Mindfulness mediates the physiological markers of stress: Systematic review and meta-analysis. Journal of Psychiatric Research, 95, 156–178. 10.1016/j.jpsychires.2017.08.004 28863392

[42] Rizvi, S. J., Pizzagalli, D. A., Sproule, B. A., & Kennedy, S. H. (2016). Assessing anhedonia in depression: Potentials and pitfalls. Neuroscience & Biobehavioral Reviews, 65, 21–35. 10.1016/j.neubiorev.2016.03.004 26959336 PMC4856554

[43] Rowa, K., Waechter, S., Hood, H. K., & Antony, M. M. (2017). Generalized Anxiety Disorder. In Psychopathology (pp. 149–186). John Wiley & Sons, Ltd. 10.1002/9781394258949.ch4

[44] Sajid, N., Ball, P. J., Parr, T., & Friston, K. J. (2021). Active Inference: Demystified and Compared. Neural Computation, 33(3), 674–712. 10.1162/neco_a_01357 33400903

[45] Sharot, T. (2011). The optimism bias. Current Biology, 21(23), R941–R945. 10.1016/j.cub.2011.10.030 22153158

[46] Sharot, T., Riccardi, A. M., Raio, C. M., & Phelps, E. A. (2007). Neural mechanisms mediating optimism bias. Nature, 450(7166), 102–105. 10.1038/nature06280 17960136

[47] Smith, R., Friston, K. J., & Whyte, C. J. (2022). A step-by-step tutorial on active inference and its application to empirical data. Journal of Mathematical Psychology, 107, 102632. 10.1016/j.jmp.2021.102632 35340847 PMC8956124

[48] Smith, R., Parr, T., & Friston, K. J. (2019). Simulating Emotions: An Active Inference Model of Emotional State Inference and Emotion Concept Learning. Frontiers in Psychology, 10. 10.3389/fpsyg.2019.02844 PMC6931387 31920873

[49] Soucy Chartier, I., & Provencher, M. D. (2013). Behavioural activation for depression: Efficacy, effectiveness and dissemination. Journal of Affective Disorders, 145(3), 292–299. 10.1016/j.jad.2012.07.023 22884236

[50] Sutton, R. S., & Barto, A. G. (2018). Reinforcement Learning, second edition: An Introduction. MIT Press.

[51] Terrill, A. L., Ruiz, J. M., & Garofalo, J. P. (2010). Look on the bright side: Do the benefits of optimism depend on the social nature of the stressor? Journal of Behavioral Medicine, 33(5), 399–414. 10.1007/s10865-010-9268-6 20563838

[52] Tindall, L., Mikocka-Walus, A., McMillan, D., Wright, B., Hewitt, C., & Gascoyne, S. (2017). Is behavioural activation effective in the treatment of depression in young people? A systematic review and meta-analysis. Psychology and Psychotherapy: Theory, Research and Practice, 90(4), 770–796. 10.1111/papt.12121 PMC5697579 28299896

